# Supplementary material for: Identifying SARS-CoV-2 antiviral compounds by screening for small molecule inhibitors of Nsp3 papain-like protease
Source: Biochem J. 2021 Jul 2;478(13):2517–31. doi: 10.1042/BCJ20210244 (PMC8286840; doi:10.1042/BCJ20210244)

Supplemental Materials

Figure S1. (Related to Figure 1) Purification of PLpro in bacteria and baculovirus.

A. Purification of His-TEV-PLpro from bacteria. Lanes 11-13 contain monomer of PLpro from the last step of the purification on a gel filtration column and these were pooled.
B. Purification of Flag-His-PLpro from baculovirus. Lane 2-4 contain monomer of PLpro from a gel filtration column and were pooled. The tag remains intact with protein.

Figure S2. (Related to Figure 2) Optimalization of the FRET assay for PLpro.

A. PLpro was able to cleave substrate Pro2 (nsp2/3) but not Pro1 (nsp1/2) (see Material & Methods)
B. PLpro was able to cleave substrate Pro3 (12 amino acids) more efficiently than Pro2 (10 amino acids).
C. Comparison between the TEV-tagged and Sumo-tagged versions of PLpro enzyme activities.
D. Comparison between the baculovirus and bacterial (TEV-tagged) purified PLpro.

Figure S3. (Related to Figure 4) Gel-based assay.

A. Purification of the substrate (67 kDa) with Superdex S200. Fractions 14-18 were pooled and used for the gel-based assay.
B. Dihydrotanshione I, beta-lapachone, cryptotanshinone, tanshinone IIA, Cdk4 inhibitor III, GLR-0617, and Ro 08-2750 did not inhibit 3CLpro (nsp5) activity. Z-VAD-FMK is a novel inhibitor discovered against nsp5 (reference nsp5 paper in this issue)

Figure S4. EC_{50} of beta-lapachone (A), Cdk4 inhibitor III (B), cryptotanshinone (C), and tanshinone IIA (D) from the cell culture-based viral proliferation assay.

Figure S5. (Related to Figure 7) (A and C) Viral proliferation assay for dihydrotanshinone I, Ro 08-2750, GRL-0617, beta-lapachone, cryptotanshinone, tanshinone IIA, and Cdk4 inhibitor III. B. There is no obvious additional effect of

combining remdesivir with compounds in (A). These compounds either do not stop COVID-19 viral proliferation or are cytotoxic.

Table S1. Compound list of over 5000 compounds with scores from low and high concentration.

Table S2. Oligomers and PLpro optimized sequence.

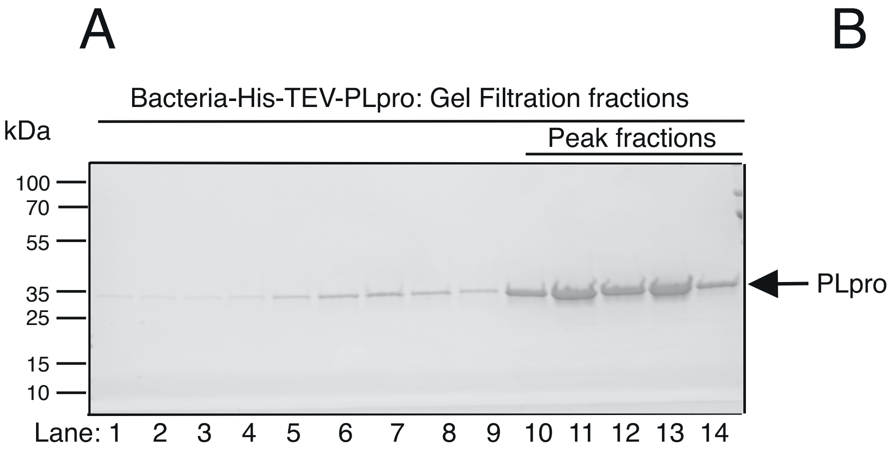
A
Bacteria-His-TEV-PLpro: Gel Filtration fractions
Peak fractions
kDa
100
70
55
35
25
15
10
PLpro
Lane: 1 2 3 4 5 6 7 8 9 10 11 12 13 14

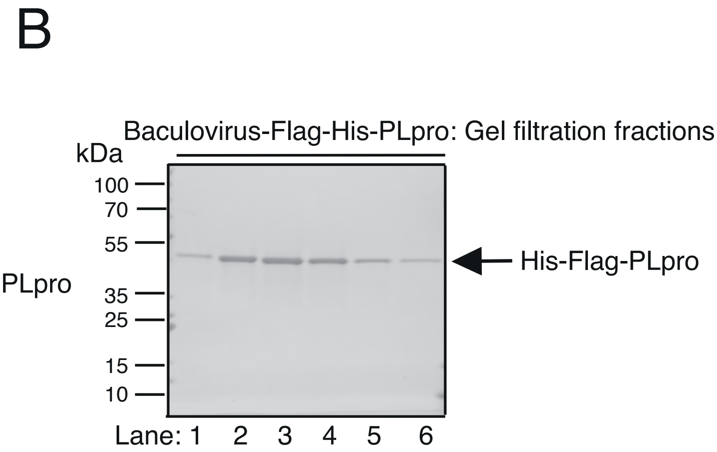
B
Baculovirus-Flag-His-PLpro: Gel filtration fractions
kDa
100
70
55
35
25
15
10
His-Flag-PLpro
Lane: 1 2 3 4 5 6

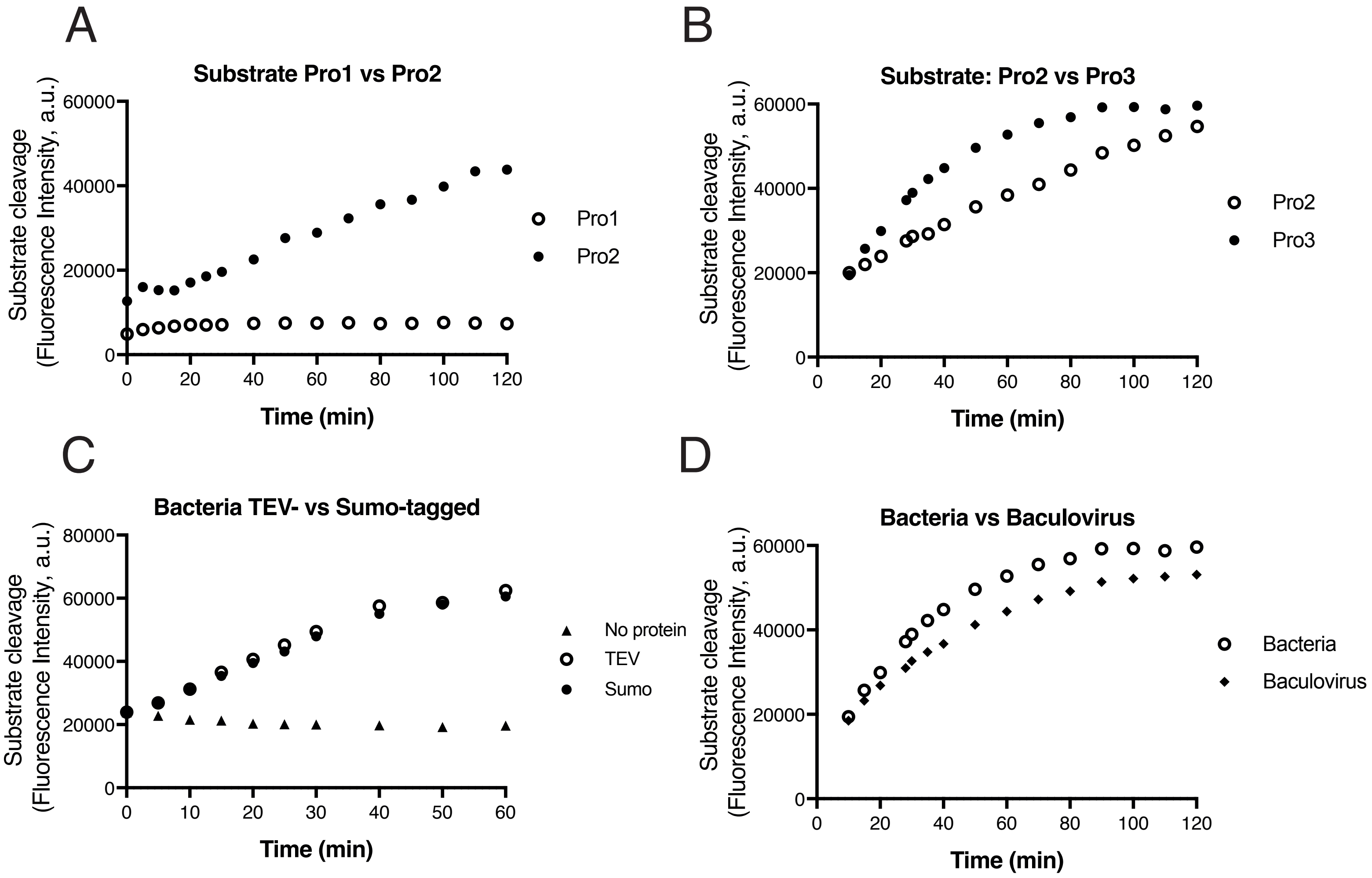
A
Substrate Pro1 vs Pro2
Substrate cleavage (Fluorescence Intensity, a.u.)
0
20000
40000
60000
0 20 40 60 80 100 120
Time (min)
Pro1
Pro2
B
Substrate: Pro2 vs Pro3
Substrate cleavage (Fluorescence Intensity, a.u.)
0
20000
40000
60000
0 20 40 60 80 100 120
Time (min)
Pro2
Pro3
C
Bacteria TEV- vs Sumo-tagged
Substrate cleavage (Fluorescence Intensity, a.u.)
0
20000
40000
60000
80000
0 10 20 30 40 50 60
Time (min)
No protein
TEV
Sumo
D
Bacteria vs Baculovirus
Substrate cleavage (Fluorescence Intensity, a.u.)
0
20000
40000
60000
0 20 40 60 80 100 120
Time (min)
Bacteria
Baculovirus

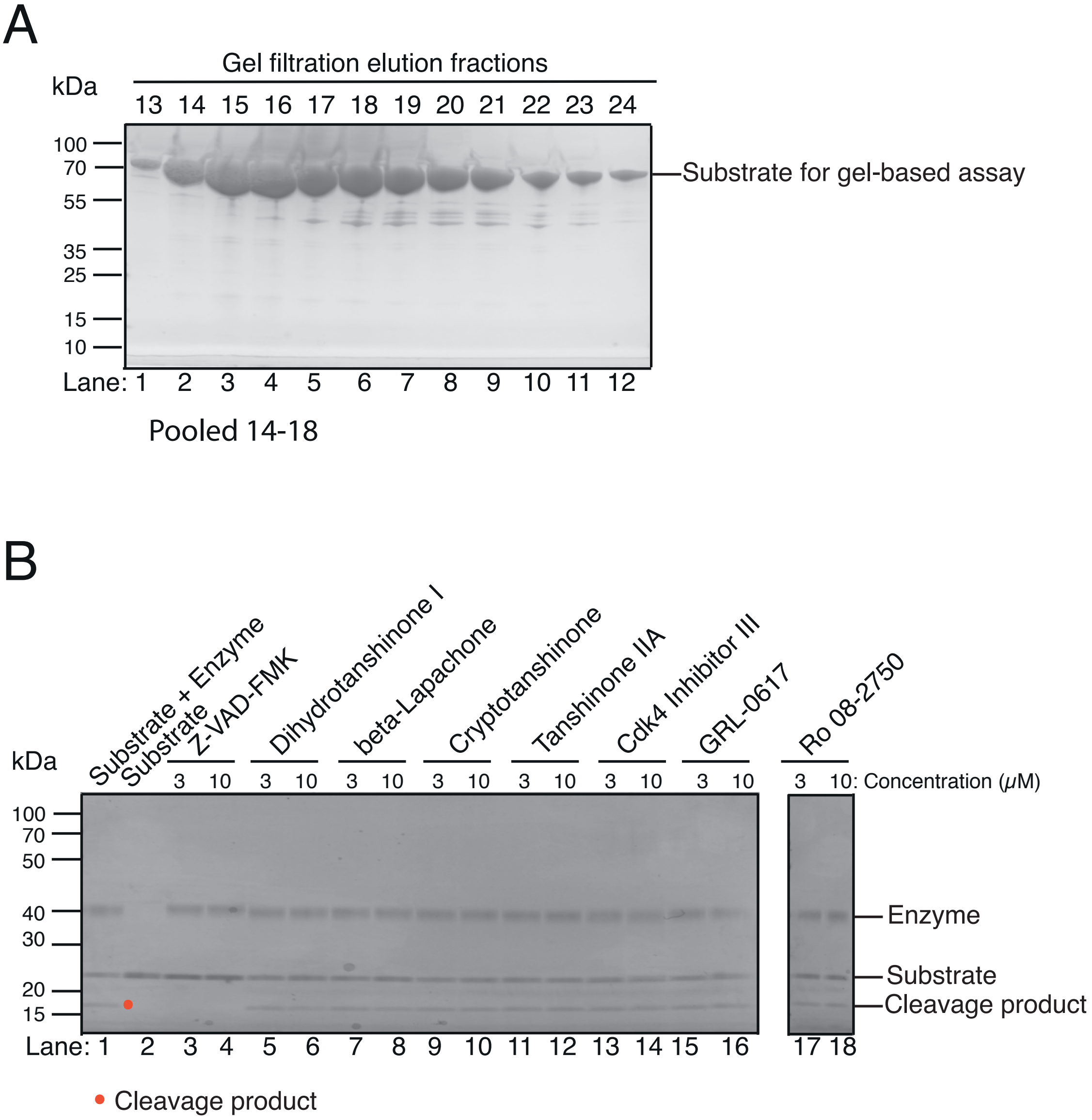
A
Gel filtration elution fractions
kDa
13 14 15 16 17 18 19 20 21 22 23 24
100
70
55
35
25
15
10
Substrate for gel-based assay
Lane: 1 2 3 4 5 6 7 8 9 10 11 12
Pooled 14-18
B
Substrate + Enzyme
Substrate
Z-VAD-FMK
Dihydrotanshinone I
beta-Lapachone
Cryptotanshinone
Tanshinone IIA
Cdk4 Inhibitor III
GRL-0617
Ro 08-2750
kDa
3 10 3 10 3 10 3 10 3 10 3 10 3 10 3 10: Concentration (μM)
100
70
50
40
30
20
15
Enzyme
Substrate
Cleavage product
Lane: 1 2 3 4 5 6 7 8 9 10 11 12 13 14 15 16 17 18
• Cleavage product

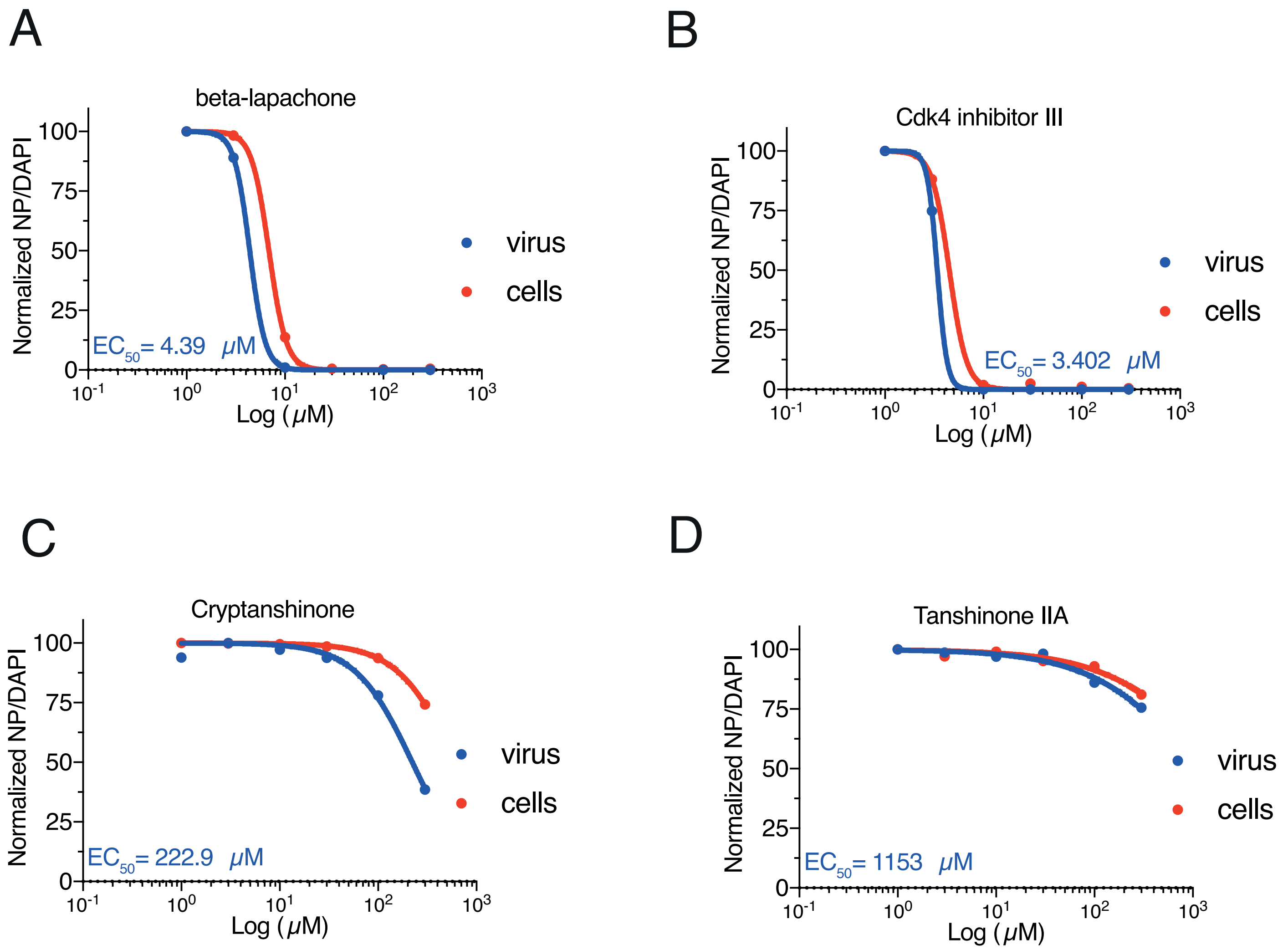
A
beta-lapachone
Normalized NP/DAPI
100
75
50
25
0
virus
cells
EC$_{50}$= 4.39 μM
10^{-1} 10^{0} 10^{1} 10^{2} 10^{3}
Log (μM)
B
Cdk4 inhibitor III
Normalized NP/DAPI
virus
cells
EC$_{50}$= 3.402 μM
Log (μM)
C
Cryptanshinone
Normalized NP/DAPI
virus
cells
EC$_{50}$= 222.9 μM
Log (μM)
D
Tanshinone IIA
Normalized NP/DAPI
virus
cells
EC$_{50}$= 1153 μM
Log (μM)

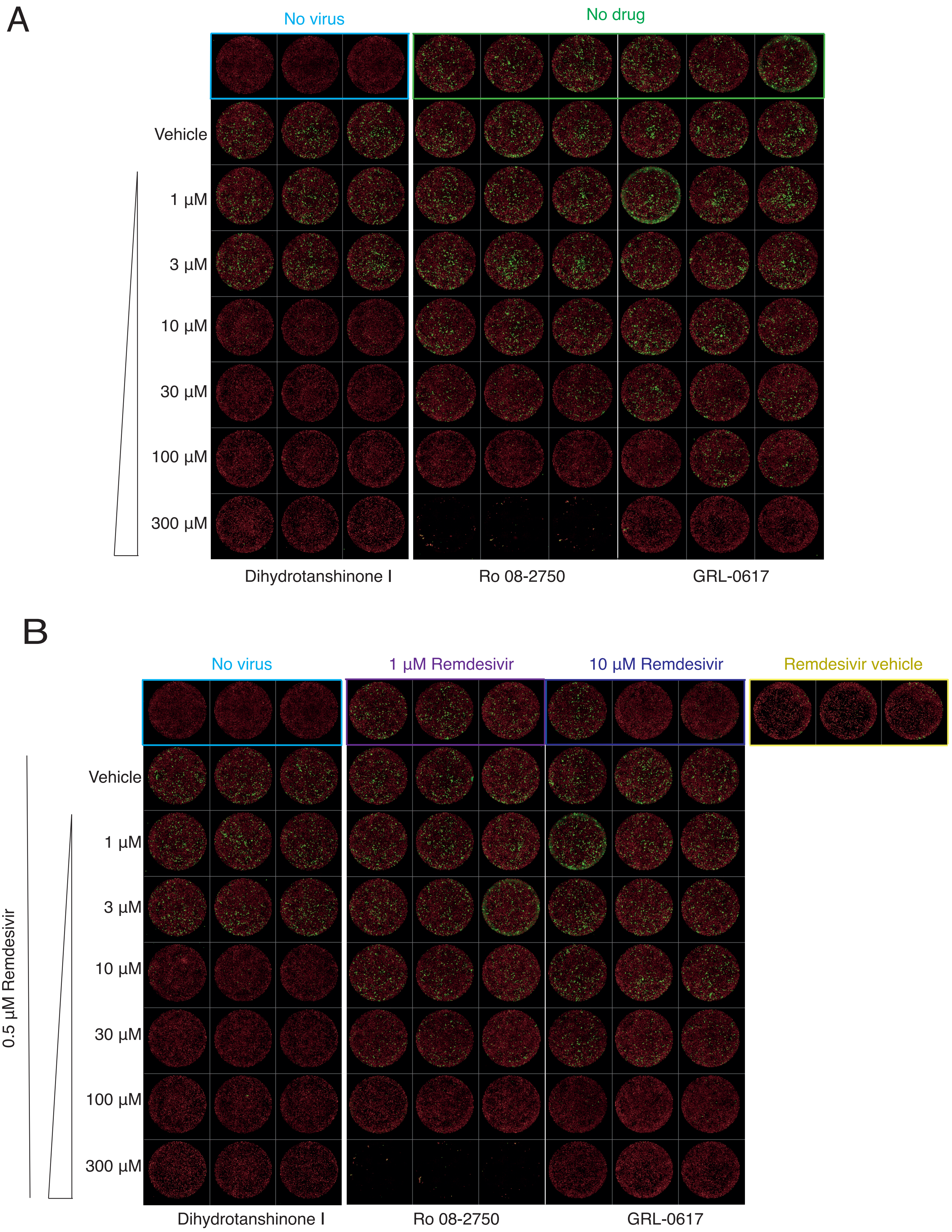
A
No virus
No drug
Vehicle
1 µM
3 µM
10 µM
30 µM
100 µM
300 µM
Dihydrotanshinone I
Ro 08-2750
GRL-0617
B
No virus
1 µM Remdesivir
10 µM Remdesivir
Remdesivir vehicle
0.5 µM Remdesivir
Vehicle
1 µM
3 µM
10 µM
30 µM
100 µM
300 µM
Dihydrotanshinone I
Ro 08-2750
GRL-0617

Supplementary Figure S5C

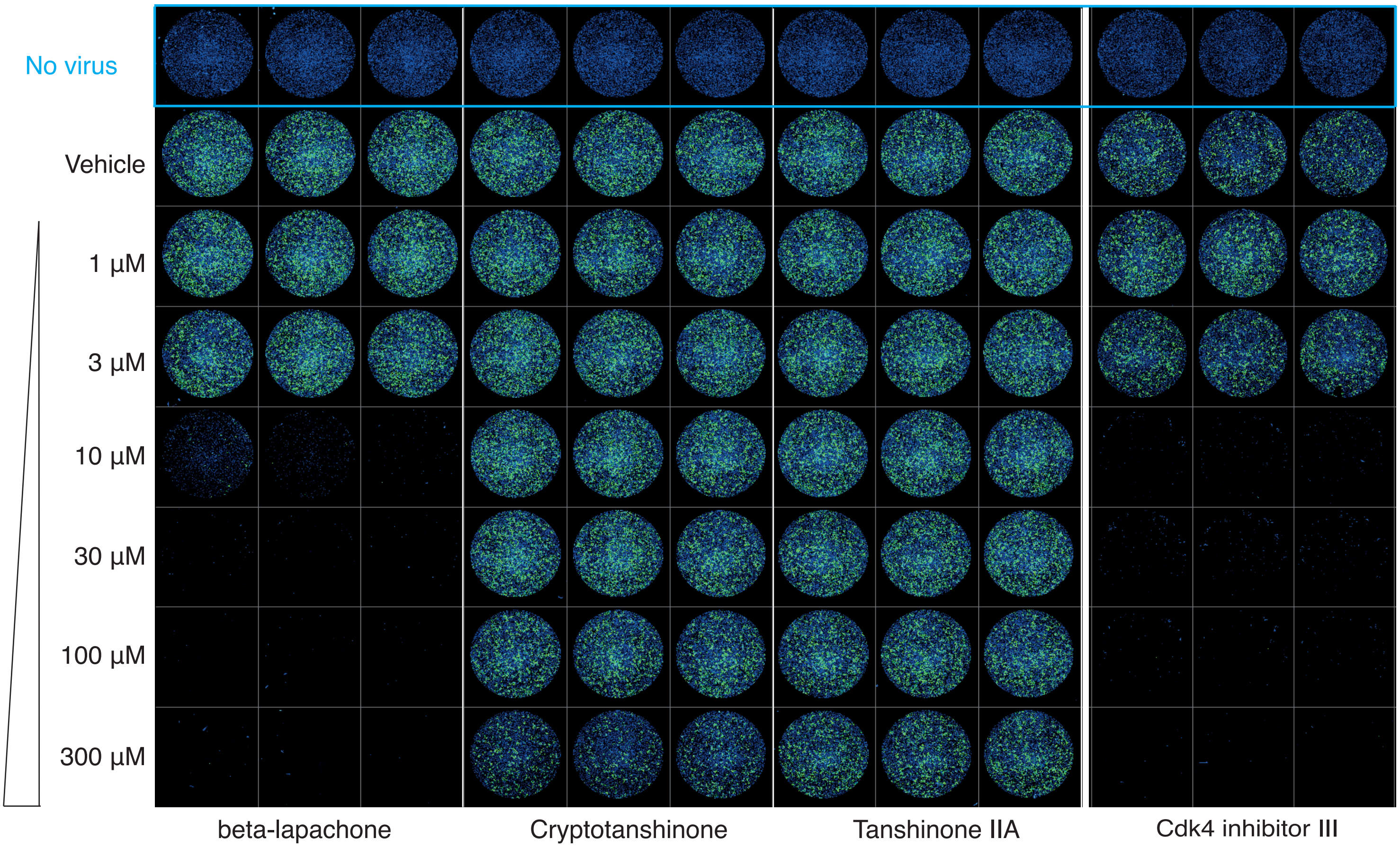

Supplement: Supplementary Figures S1-S5 [file BCJ-478-2517-s1.pdf]
